# Transcriptome and Proteome Expression Analysis of the Metabolism of Amino Acids by the Fungus *Aspergillus oryzae* in Fermented Soy Sauce

**DOI:** 10.1155/2015/456802

**Published:** 2015-04-07

**Authors:** Guozhong Zhao, Yunping Yao, Chunling Wang, Fengwei Tian, Xiaoming Liu, Lihua Hou, Zhen Yang, Jianxin Zhao, Hao Zhang, Xiaohong Cao

**Affiliations:** ^1^Key Laboratory of Food Nutrition and Safety (Tianjin University of Science & Technology), Ministry of Education, Tianjin 300457, China; ^2^State Key Laboratory of Food Science and Technology, School of Food Science and Technology, Jiangnan University, 1800 Lihu Road, Wuxi, Jiangsu 214122, China

## Abstract

Amino acids comprise the majority of the flavor compounds in soy sauce. A portion of these amino acids are formed from the biosynthesis and metabolism of the fungus *Aspergillus oryzae*; however, the metabolic pathways leading to the formation of these amino acids in *A. oryzae* remain largely unknown. We sequenced the transcriptomes of *A. oryzae* 100-8 and *A. oryzae* 3.042 under similar soy sauce fermentation conditions. 2D gel electrophoresis was also used to find some differences in protein expression. We found that many amino acid hydrolases (endopeptidases, aminopeptidases, and X-pro-dipeptidyl aminopeptidase) were expressed at much higher levels (mostly greater than double) in *A. oryzae* 100-8 than in *A. oryzae* 3.042. Our results indicated that glutamate dehydrogenase may activate the metabolism of amino acids. We also found that the expression levels of some genes changed simultaneously in the metabolic pathways of tyrosine and leucine and that these conserved genes may modulate the function of the metabolic pathway. Such variation in the metabolic pathways of amino acids is important as it can significantly alter the flavor of fermented soy sauce.

## 1. Introduction

Soy sauce, the most popular condiment in Asian countries, is fermented by the fungus* Aspergillus oryzae *and yeasts. During the fermentation and ageing, the flavor may develop gradually. Soy sauce contains many flavor compounds, including alcohols, aldehydes, esters, and acids; however, amino acids form the main substance of the flavor of soy sauce. Amino acid nitrogen is one of the most important indicators of soy sauce quality, which determines the quality grade of soy sauce in China [1]. Amino acids are the building blocks of proteins and can be produced via enzymatic hydrolysis of proteins.* A. oryzae* is equipped with powerful enzyme systems that can both break down proteins into amino acids and synthesize amino acids during metabolism to produce flavor compounds [2]. Indeed, microbial catabolism of amino acids produces important flavor compounds in soy sauce.

The metabolic pathways leading to the formation of amino acids in* A. oryzae* remain largely unknown. However, the recent sequencing of the genome of* A. oryzae* 100-8, which secretes more acid proteases and grows faster than the original strain of* A. oryzae* 3.042, has opened the door to investigations in this area [3]. In this study, we utilize this genomic sequence to quantify gene expression levels of the amino acid metabolism pathways within* A. oryzae* 100-8 and 3.042 via the analysis of the transcriptome and the proteome.

## 2. Materials and Methods

### 2.1. Strains

The wild-type strain* A. oryzae* 3.042 and the mutant strain* A. oryzae* 100-8 were obtained from the Strain Collection Center of Tianjin University of Science & Technology (China). Strains were cultivated at 28°C for 30 h, 36 h, and 42 h, and the mycelia of the two strains were collected for the preparation of RNA-Seq and protein sequencing procedures.

### 2.2. Transcriptome Sequencing and Analysis

To extract mRNA, the samples of* A. oryzae* 100-8 and 3.042 were frozen in liquid nitrogen and then treated with TRIzol solution, DNaseI, and Sera-mag Magnetic Oligo (dT) Beads (Illumina) according to the manufacturers' protocols [4]. The cDNA libraries were generated according to the Massively Parallel Signature sequencing protocol after reverse transcription. The cDNA was end-repaired, amplified, denatured, and then sequenced with an Illumina Genome Analyzer IIx using proprietary reagents. RNA-Seq libraries were constructed using SOLiD Total RNA-Seq Kit, and the reads were mapped to the genomes of* A. oryzae *100-8 and 3.042. Gene expression levels were measured in terms of “fragments per kilobase of exon model per million mapped reads” (FPKM values).

### 2.3. 2-DE: Protein Identification and Bioinformatics Analysis

The 2-DE experiment was performed according to the MIAPE standards [5]. Mycelia were ground into powder with liquid nitrogen and treated with lysis buffer, 8 M urea, 2 M thiourea, 0.5% (wt/vol) CHAPS, 2% (wt/vol) Pharmalyte, 1% (wt/vol) DTT, 1 mM PMSF, 50 *µ*g/mL DNase I, and 50 *µ*g/mL RNase. The protein solution was mixed with rehydration buffer. IPG strips (17 cm, pH between 4 and 7) were used for initial electrophoresis, and then the equilibrated strips were run on 12% SDS-polyacrylamide gels (26 cm × 20 cm). Proteins were visualized with a staining solution. Stained protein spots were excised from the gels and the stain was removed. They were then vacuum-dried and digested with 8 *µ*L trypsin solution before being analyzed with a 4700 Proteomics Analyzer. Peptide sequences of the protein spots were determined by BLAST and SEARCH against the NCBI website and the InterProScan database [6].

## 3. Results and Discussion

### 3.1. Amino Acid hydrolase

During the fermentation of soy sauce, amino acids are released in a multistep procedure that hydrolyzes the proteins. Different parts of the peptides are hydrolyzed to amino acids by intracellular peptidases (e.g., endopeptidases, aminopeptidases, and X-pro-dipeptidyl aminopeptidase) [7]. In this study, we found that, between 30 and 42 h incubation, the expression of aspartic-type endopeptidase was greater (2.6 to 7.5 times greater) in* A. oryzae* 100-8 than in* A. oryzae* 3.042. Aminopeptidase hydrolases catalyze the removal of amino-terminal amino acids from proteins. We also found that at every stage the genes of dipeptidyl aminopeptidase (AO1008_08925) and X-pro aminopeptidase (AO1008_00627) were expressed at double the expression levels in* A. oryzae* 100-8 compared to that in* A. oryzae* 3.042. In addition, the expression of dipeptidyl aminopeptidase in* A. oryzae* 100-8 was 8.6 times greater in* A. oryzae* 3.042 at 42 h than in* A. oryzae* 3.042. The amino acids were hydrolyzed into the fermentation broth of soy sauce, with some of the amino acids being used in further microbial metabolism (Table S1).

### 3.2. Amino Acid Transporter

The amino acid transport system has been proposed to be one of the major nutrient transport systems [8]. The transport of molecules comprised of different kinds of amino acids across cell membrane is of major importance for the formation of the flavor compounds. This transport of amino acids, like that of a number of other compounds, requires the action of transporters. In this study, we observed that there was an approximate 2-fold increase in the expression level of amino acid transporters in* A. oryzae* 100-8. This is likely to cause significant differentiation in modulating transmembrane signaling (Table S1).

### 3.3. Amino Acid Metabolism

**Figure 1:**
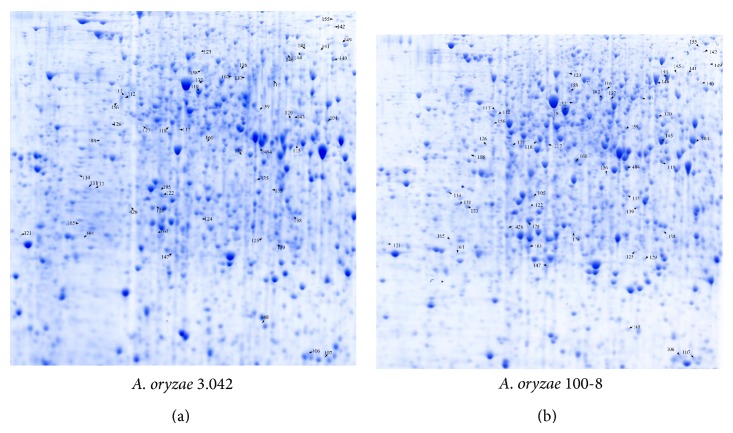
The proteome analysis of* A. oryzae* 3.042 and 100-8 grown for 36 hours (pI 4–7) in amino acid catabolism. Different expressed proteins are marked by arrows and numbers and also listed in Table S2 (see Supplementary Material available online at http://dx.doi.org/10.1155/2015/456802).

Branched-chain amino acids (valine, leucine, and isoleucine), aromatic amino acids (tyrosine, tryptophan, and phenylalanine), and sulfuric amino acids (methionine and cysteine) can be converted into flavor compounds by microbial metabolism. Branched-chain amino acids can be converted into specific malty, fruity, buttery, and sweaty flavors. Aromatic amino acids can be catabolized into compounds contributing to flavors like rose, flowers, and even bitter almond. Meanwhile, sulfuric amino acids contribute to the flavors found in potatoes, meat, and egg [9]. In this study, we found a twofold increase in the expression level of the majority of the enzymes that we previously identified as being part of amino acid biosynthesis (proteome analysis) [10] in* A. oryzae* 100-8 (Figure 1, Table S2).

Catabolism of amino acids starts with the removal of the amino group by aminotransferases. At this stage, *α*-keto acids are required as the amino group acceptors in transamination. The specific *α*-keto acids can be produced either from different kinds of amino acids or via the TCA cycle, such as *α*-ketoglutarate. In this study, we found that the ratio of expression level of glutamate dehydrogenase spots (104, 117, 118, 119, 120, 121, 122, 127) to all the spots on 2-DE gel in* A. oryzae* 3.042 was 0.7%, while the same ratio in* A. oryzae* 100-8 was 0.27% [10]. The transcriptome data revealed that expression of glutamate dehydrogenase (AO1008_09334) in* A. oryzae* 100-8 increased approximately 2.4 to 2.8 times at 36 h and 42 h. Glutamate dehydrogenase promotes the interconversion between *α*-ketoglutarate and glutamate. Glutamate is then converted into the “glutamate family” of amino acids (arginine, ornithine, proline, histidine, and glutamine) for metabolic disposal [11].

**Figure 2:**
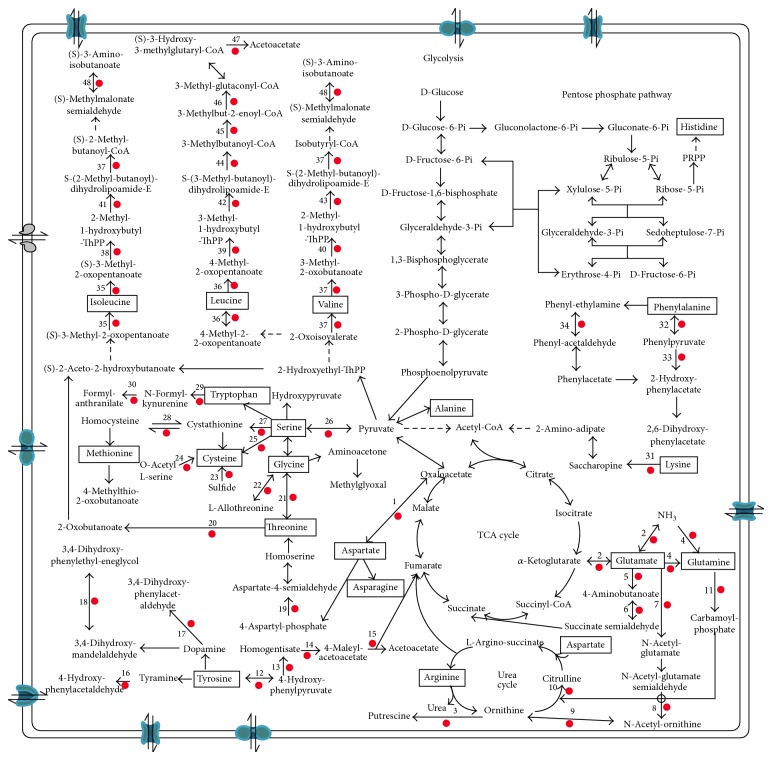
The pathways of amino acid catabolism. Those genes that were expressed at a >2-fold greater level in* A. oryzae* 100-8 than in* A. oryzae* 3.042 are indicated by red circles. The gene expression levels are listed in Table S1.

As shown in Figure 2, we found that the enhanced expression of enzymes in the metabolic pathways of tyrosine, valine, leucine, and isoleucine promoted the generation of acetoacetates (Tables S1 and S3). The aspartic acid and glutamate were metabolized to succinic acid and oxaloacetic acid, respectively, before entering the TCA cycle. In microorganisms during the metabolism of aspartic acid, aspartate semialdehyde dehydrogenase (AO1008_11523) produces the branch point intermediate between the lysine and the threonine/methionine pathways [12]. Lysine is metabolized to acetyl-CoA. The phenylpyruvate can be formed by phenylalanine, and pyruvate is formed by serine, cysteine, and methionine. Pyruvate plays a central role in regulating the balance of the tricarboxylic acid (TCA) cycle of energy metabolism and amino acid metabolism and is, thus, critically important for cellular homeostasis and metabolism.

### 3.4. Tyrosine and Leucine Metabolism

**Figure 3:**
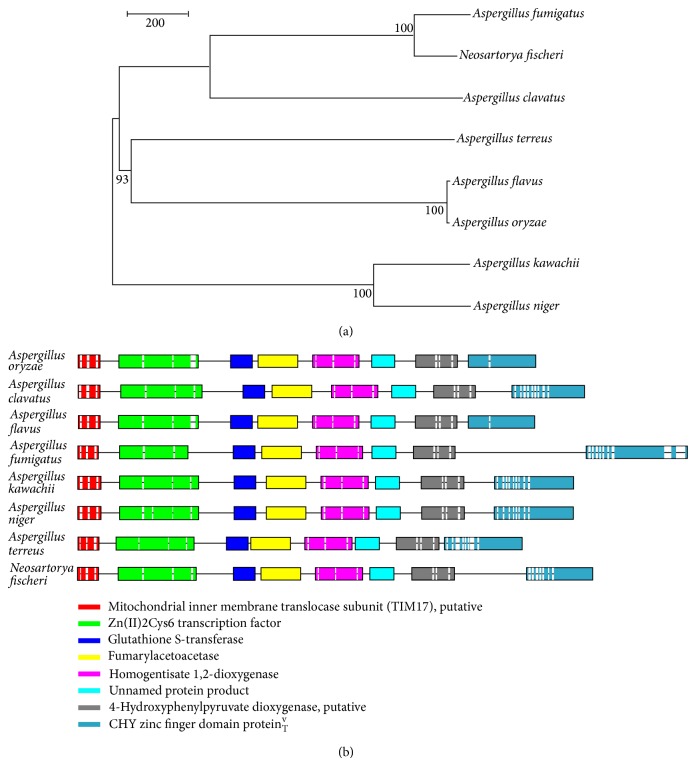
Comparison of the (a) evolutionary relationships and (b) gene sequences of* A. oryzae, A. clavatus, A. flavus, A. fumigates, A. kawachii, A. niger, A. terreus, *and* Neosartorya fischeri *in terms of the catabolic pathway of tyrosine.

**Figure 4:**
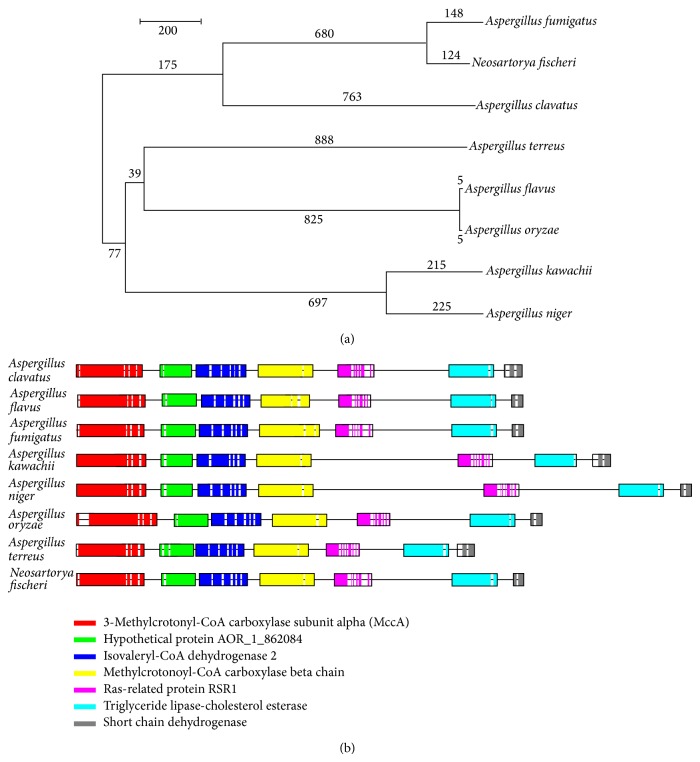
Comparison of the (a) evolutionary relationships and (b) gene sequences of* A. oryzae*,* A. clavatus*,* A. flavus*,* A. fumigates*,* A. kawachii*,* A. niger*,* A. terreus,* and* Neosartorya fischeri* in terms of the catabolic pathway of leucine.

We found that the expression levels of the related enzymes in the metabolism of tyrosine and leucine increased simultaneously. A comparison of thegene sequences of* A. oryzae* in the tyrosine and leucine metabolism pathways with those of closely related species (*A. clavatus, A. flavus, A. fumigates, A. kawachii, A. niger, A. terreus, and Neosartorya fischeri*) revealed their similar genetic structures (Figures 3 and 4). The enzyme 3-methylcrotonyl-CoA carboxylase catalyzes the carboxylation of the 3-methyl groups of homologous acyl-CoA acceptors, and isovaleryl-CoA dehydrogenase (AO1008_00532) catalyzes the third step in the catabolism of leucine [13].

**Figure 5:**
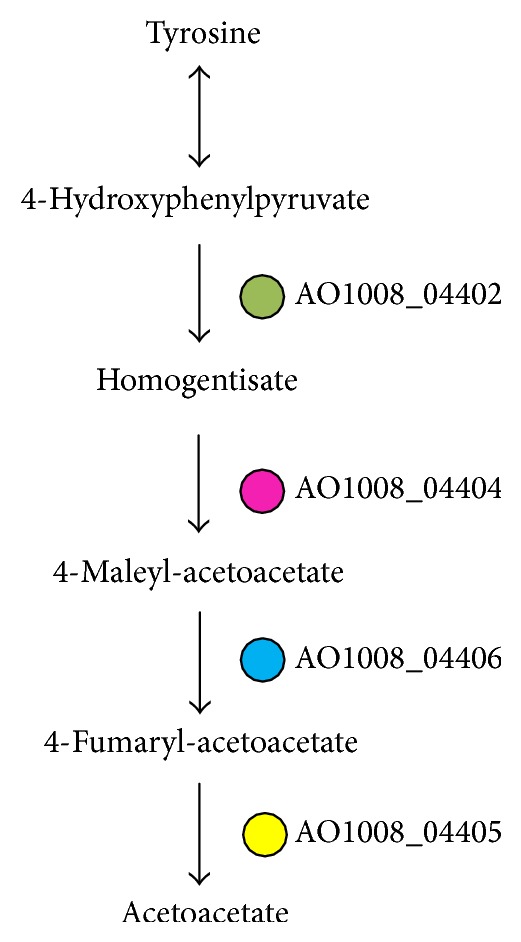
The genes and metabolites of the catabolic pathway of tyrosine that were expressed at a >2-fold greater level in* A. oryzae* 100-8 than in* A. oryzae* 3.042.

The enzyme 4-hydroxyphenylpyruvate dioxygenase (AO1008_04402) is a nonhemeoxygenase that catalyzes the conversion of 4-hydroxyphenylpyruvate to homogentisate [14]. Meanwhile, homogentisate 1,2-dioxygenase (AO1008_04404) is an iron containing enzyme that catalyzes the conversion of homogentisate to 4-maleylacetoacetate [15]. Glutathione S-transferase (AO1008_04406) is predominantly expressed in the cytosol, reducing the isomerization of maleylacetoacetate to fumarylacetoacetate, a key step in the catabolism of tyrosine [16]; see Figure 5. Fumarylacetoacetase (AO1008_04405) is the final enzyme of the metabolism pathway of tyrosine [17]. The genes of the Zn-finger protein (AO1008_04401), Zn(II)2Cys6 transcription factor (AO1008_04407), and C6 transcription factor (AO1008_04408) as the additional conserved genes may modulate the function of the metabolic pathway. Such variation in the metabolic pathways of amino acids is important as it can significantly alter the flavor of fermented soy sauce.

Overall, amino acids are the majority of the flavor compounds in soy sauce. The expression levels of genes in amino acid metabolism pathways were compared and analyzed between* A. oryzae* 100-8 and 3.042. Some differences were presented, such as the amino acid hydrolase, amino acid transporter, and amino acid metabolism. This study provided a global view of comparison of the transcriptomes and proteomes of two* A. oryzae* strains and may help us to develop strategies to improve the production of amino acids in the soy sauce fermentation industry.

## Supplementary Material

Table S1: The FPKM values of all the genes and genes that involved in amino acid metabolism in A. oryzae 100-8 and 3.042, along with a comparison of those that are related in the two fungus strains.
Table S2: The identified spots related to amino acid metabolism of the proteome analysis of A. oryzae 3.042 and 100-8 applying 2D PAGE and MALDI-TOF MS.
Table S3: Corrsponding enzymes (coding genes) of biochemical reactions potentially catalyzing these reactions. Reactions and CDSs depicted in Figure 2 are indicated.
